# Social Inequality and Structural Vulnerability as Predictors of Health-Related Quality of Life: A Prospective Cohort Study

**DOI:** 10.7759/cureus.116529

**Published:** 2026-09-19

**Authors:** Madiha Irfan

**Affiliations:** 1 Public Health Dentistry, Liaquat University of Medical & Health Sciences, Karachi, PAK

**Keywords:** cohort studies, health status disparities, quality of life, social determinants of health, structural vulnerability

## Abstract

Background

Social inequality and structural vulnerability are increasingly recognized as important social determinants of health; however, longitudinal evidence regarding their impact on health-related quality of life (HRQoL) remains limited, particularly in low- and middle-income countries. This study explored associations between social inequality, structural vulnerability, and HRQoL over a six-month follow-up period in adults in Pakistan.

Methods

We conducted a prospective cohort study of 600 adult participants recruited through healthcare facilities and community-based settings in Karachi, Pakistan, using non-probability convenience sampling. Baseline assessments comprised a composite social inequality index, the Structural Vulnerability Assessment Tool (SVAT), and the World Health Organization Quality of Life-BREF (WHOQOL-BREF) questionnaire. We reassessed HRQoL after six months, with 593 participants completing follow-up. We used Spearman correlation analysis, linear mixed-effects modelling, and analysis of covariance to examine associations between social inequality, structural vulnerability, and HRQoL.

Results

Higher social inequality was significantly associated with lower HRQoL at baseline (ρ = -0.530, p < 0.001), and higher structural vulnerability was significantly associated with lower HRQoL at baseline (ρ = -0.691, p < 0.001). Quality of life(QoL) declined significantly over six months (B = -6.09, p < 0.001). Structural vulnerability (B = -1.48, p < 0.001) and chronic disease burden (B = -2.08, p < 0.001) independently predicted poor HRQoL over time. However, after adjustment, structural vulnerability remained an independent predictor of HRQoL six months later, whereas social inequality was not.

Conclusion

Structural vulnerability was the strongest independent predictor of HRQoL, underscoring the need to address structural determinants of health and well-being alongside clinical care to minimize health inequities and improve HRQoL over time.

## Introduction

Health-related quality of life (HRQoL) is a multifaceted concept that reflects an individual's physical, psychological, and social functioning and is increasingly regarded as a key determinant of health [1, 2]. Although medical care is an important factor in health outcomes, growing evidence shows that social determinants of health, including socioeconomic inequalities, significantly affect an individual's health-related quality of life [3, 4].

Social inequality, defined as unequal access to income, education, employment, and health services, reduces opportunities to achieve optimal health. At the same time, structural vulnerability is associated with the combined risk of poor health stemming from social, economic, and institutional disadvantages [5, 6]. People living in poor social circumstances face a higher likelihood of psychological distress and diminished HRQoL [4, 7].

However, previous research has shown strong links between socioeconomic disadvantage and poorer HRQoL, with most studies examining social inequality and structural vulnerability in isolation and with mostly cross-sectional designs, which do not allow for causal inferences [4, 8]. While social inequality and structural vulnerability have been linked to health outcomes, longitudinal evidence on their effects on HRQoL remains limited; most existing studies use cross-sectional designs [9, 10].

Thus, a prospective cohort design is a better methodological approach for studying the association of baseline social conditions with later HRQoL. The present study is novel in that it simultaneously examines social inequality and structural vulnerability as predictors of HRQoL over time, using standardized measurement instruments in a Pakistani population.

This study aimed to examine associations between social inequality and structural vulnerability and HRQoL in a prospective cohort of adults. Specifically, the study aimed to assess baseline levels of social inequality, structural vulnerability, and HRQoL; evaluate the associations between these social determinants and subsequent HRQoL; and identify whether social inequality and structural vulnerability independently predict changes in HRQoL after adjusting for relevant demographic and socioeconomic characteristics. The results will support evidence-based public health strategies and policies to reduce health inequities and enhance quality of life in vulnerable populations.

## Materials and methods

Study design

This prospective cohort study investigated associations between social inequality, structural vulnerability, and HRQoL in adults. We assumed social inequality indicators and structural vulnerability as baseline exposure variables and measured them only at enrolment. HRQoL was considered the study outcome and was reassessed after six months to evaluate longitudinal changes.

Study setting

We collected data from public and private health care facilities and community-based sites in Karachi, Pakistan. To ensure adequate representation across socioeconomic backgrounds, participants were recruited from outpatient departments and community health centers. We collected data between July 2025 and May 2026.

Study population

Adults aged 18 years or older who could understand English or Urdu and were willing to provide written informed consent. Severe cognitive impairment, active psychiatric illness that prevented a person from completing questionnaires, terminal illness, and inability to participate in follow-up assessment were excluded.

Sample size and sampling technique

The sample size was calculated using Cochran's formula for large populations, with a 95% confidence level, a 5% margin of error, and an assumed population proportion of 50%, resulting in a required minimum sample size of 384 participants. To increase statistical power, improve estimate precision, and account for potential loss to follow-up during the six-month study period, we recruited and enrolled 600 participants at baseline. At the end of the six-month follow-up, 593 participants completed the final assessment, while seven participants were lost to follow-up. Therefore, the final longitudinal analysis included 593 individuals.

We used a non-probability convenience sampling technique. During the study period, eligible participants were approached consecutively at the selected healthcare facilities and invited to participate until the required sample size of 600 was reached.

Data collection instruments

Demographic Questionnaire (Social Inequality)

We assessed social inequality using a structured demographic questionnaire developed after reviewing the literature on social determinants of health. Social inequality was operationalized through several socioeconomic indicators such as age, gender, marital status, level of education, employment status, occupation, monthly household income, residence, housing status, household size, health insurance status, smoking status, alcohol consumption, presence of chronic diseases, number of financially dependent family members, and principal sources of family income.

We generated a composite social inequality index by coding each socioeconomic indicator so that higher values represented greater social disadvantage. We included education, employment status, household income, housing status, health insurance status, chronic disease status, and other demographic characteristics as indicators of social inequality. Each coded variable was equally weighted in the index, and the mean of the coded scores was computed to create an overall social inequality index for each participant.

For reproducibility, we coded categorical indicators numerically before aggregation. Gender was coded as male = 1 and female = 2; marital status as single = 1, married = 2, divorced = 3, widowed = 4, and separated = 5; residence as urban = 1 and rural = 2; education as no formal education = 1, primary education = 2, secondary education = 3, higher secondary = 4, bachelor’s degree = 5, master’s degree = 6, and MPhil/PhD = 7; employment status as full-time employed = 1, part-time employed = 2, self-employed = 3, unemployed = 4, student = 5, homemaker = 6, and retired = 7; occupation as professional/managerial = 1, clerical/office = 2, business owner = 3, manual/labor = 4, and service/sales = 5; and monthly household income as < Pakistani Rupees (PKR) 30,000 = 1, PKR 30,000-59,999 = 2, PKR 60,000-99,999 = 3, PKR 100,000-149,999 = 4, and ≥ PKR 150,000 = 5. Binary indicators were coded as 0 = no and 1 = yes.

Equal weighting was selected to provide a transparent, non-subjective approach, as no empirically validated weighting scheme for these indicators exists in the study population. This approach ensured that no single socioeconomic or social disadvantage indicator disproportionately influenced the overall index score. The resulting index was therefore intended as a pragmatic multidimensional measure of social disadvantage rather than a validated psychometric scale. A high index score represented a high level of social disadvantage. All other statistical analyses were conducted on this composite score. Selected indicators were drawn from a set of social determinants of health identified in the literature and used to create a multi-dimensional measure of social inequality rather than a socioeconomic indicator [3, 5].

Structural Vulnerability Assessment Tool (SVAT)

The SVAT, created by Bourgois et al., was used to assess structural vulnerability. The tool assesses social and structural disadvantages across various areas, including financial security, housing and residence, food access, social support, risk environment, legal status, education and health literacy, and experiences of discrimination. High scores denote high structural vulnerability and exposure to adverse social conditions. The SVAT was developed as a structured clinical assessment instrument to evaluate multiple domains of structural vulnerability and has been widely adopted to operationalize it in health research and clinical contexts [6].

World Health Organization Quality of Life-BREF (WHOQOL-BREF) 

HRQoL was measured using the WHOQOL-BREF questionnaire (1998) developed by the World Health Organization Quality of Life Group. WHOQOL-BREF contains 26 items, including two general quality-of-life questions; physical health domain (seven items); psychological domain (six items); social relationships domain (three items); and environment domain (eight items). The items are scored on a 5-point Likert scale from 1 to 5. We derived raw domain scores using the WHO scoring guidelines and converted them to a 0-100 range, with higher scores indicating better perceived HRQoL. Previous studies have shown acceptable to good internal consistency (Cronbach's alpha coefficients ranging from 0.66 to 0.84) for all four domains. Baseline HRQoL was assessed at enrolment and re-evaluated at six months post-enrolment using the same instrument [11].

Data collection procedure

After Institutional Review Board approval, eligible participants were identified at the selected healthcare facilities and community settings. All participants gave written informed consent before entering the study, which was conducted in accordance with its objectives and procedures and involved potential benefits and risks. The baseline assessment comprised the demographic questionnaire, the SVAT, and the WHOQOL-BREF. For participants who could not understand English, trained research assistants verbally explained the instructions and questionnaire items in Urdu during the interview; no formal Urdu translation or validated Urdu version of the questionnaires was used. Research assistants provided clarification when necessary without influencing participants' responses and recorded the participants' answers. The same standardized administration procedure was followed at baseline and at the six-month follow-up. We collected contact details to enable follow-up. After six months, researchers contacted participants by telephone to arrange appointments to administer the WHOQOL-BREF questionnaire and assess changes in HRQoL during the follow-up period. We checked all returned questionnaires for completeness and accuracy before data entry and analysis.

Statistical analysis

We analyzed the data using SPSS Statistics version 26.0 (IBM Corp., Armonk, USA). We used the Shapiro-Wilk test and visual inspection to assess normality. We summarized continuous variables as mean ± SD or median (IQR) and categorical variables as frequencies and percentages. Correlation analysis (Spearman) was used to estimate the associations between the social inequality index and the total score of the HRQoL and SVAT.

We assessed the effects of time on HRQoL using a linear mixed-effects model with a random intercept for participant ID and repeated measures of time. We used the linear mixed-effects model and analysis of covariance (ANCOVA) as complementary analyses because they addressed different aspects of the longitudinal data. The linear mixed-effects model assessed changes in HRQoL over time while accounting for repeated measurements within participants and examined whether associations between social inequality and structural vulnerability and HRQoL changed over the six-month follow-up.

ANCOVA assessed six-month HRQoL after adjustment for baseline HRQoL and relevant covariates. We selected covariates a priori based on their potential associations with HRQoL and social vulnerability, including age, gender, residence, and number of chronic diseases. Fixed effects included time, social inequality index, SVAT total score, age, gender, residence, and number of chronic diseases. We included interaction terms between time and the social inequality index and between time and the SVAT total score to determine whether changes in HRQoL over the six-month follow-up differed by baseline social inequality or structural vulnerability, respectively.

To assess the robustness of the social inequality index, we conducted sensitivity analyses using alternative scoring approaches. These included removing individual index components, standardizing component indicators before aggregation, categorizing the index into levels of inequality, and applying alternative weighting assumptions. We compared estimates from these models with the primary equally weighted index model. Results were presented as B, SE, 95% CI, and p-values. At baseline, 600 participants were enrolled; 592 participants had complete data for the variables included in the Spearman correlation analysis, with eight participants having missing responses in one or more of these variables (1.3%). We handled these missing observations using pairwise deletion for the correlation analyses to maximize available information for each correlation. Seven participants (1.2%) were lost to follow-up, while 593 participants completed the six-month assessment. The mixed-effects model used all available repeated-measures data under the missing-at-random assumption. A p-value < 0.05 was considered statistically significant.

Ethical considerations

Before data collection, the study received ethical approval from the Institutional Review Board of Liaquat University of Medical and Health Sciences, Jamshoro, DOC #LUMH/IRB/R-1-551/72. We obtained written informed consent after explaining the study's aims, procedures, risks, and benefits to all participants. Participation was voluntary, and participants were told they could leave the study at any time without repercussions. The researchers ensured confidentiality and anonymity by assigning unique identification numbers to each participant, securely storing all collected data, and making it accessible only to the research team. The research adhered to the ethical principles outlined in the Declaration of Helsinki.

## Results

Figure 1 shows the flow of participants during the study. At baseline, 600 participants were enrolled. A total of 593 participants (98.8%) completed the follow-up assessment and were included in the final longitudinal analyses, while seven participants (1.2%) were lost to follow-up.

**Figure 1:**
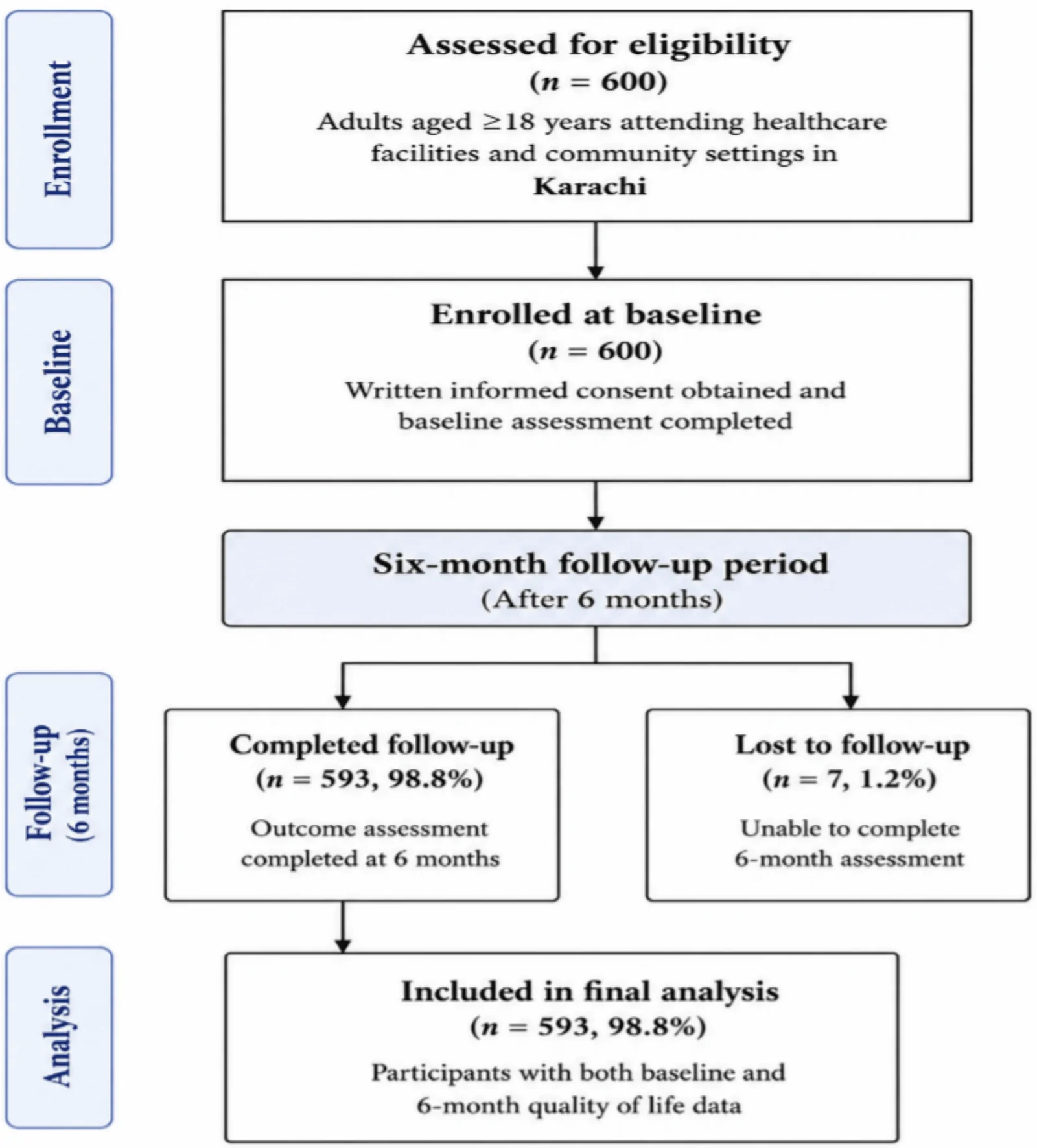
Participant Flow Diagram

Table 1 presents the descriptive statistics of the continuous study variables for the 600 participants. The mean age of the participants was 41.52 ± 11.84 years, ranging from 19 to 75 years. The mean household size was 4.27 ± 1.91 individuals (range: 1-12). The number of chronic diseases reported by participants was 0.22 ± 0.59, ranging from 0 to 4. The average number of financial dependents was 2.52 ± 1.69 (range: 0 to 10). The mean social inequality index score was 44.59 ± 21.19, with scores ranging from 0.00 to 91.50, reflecting significant inter-individual differences in social disadvantage among the participants.

**Table 1:** Descriptive Statistics of Continuous Study Variables (N = 600) Values are presented as Mean ± SD for continuous variables. Higher scores on the social inequality index indicate greater social disadvantage; N=600.

Variable	Mean ± SD	Min.	Max.
Age (years)	41.52 ± 11.84	19	75
Household size (number of people)	4.27 ± 1.91	1	12
Number of chronic diseases	0.22 ± 0.59	0	4
Financial dependents	2.52 ± 1.69	0	10
Social inequality index	44.59 ± 21.19	0.00	91.50

Sociodemographic, economic, and health characteristics of the 600 participants at baseline are shown in Table 2. Females (n = 325, 54.2%) slightly outnumbered males (n = 275, 45.8%), and most participants were married (n = 336, 56.0%) and resided in urban areas (n = 401, 66.8%). The most common educational level was primary education (n = 124, 20.7%), while the most prevalent employment categories were full-time employment (n = 165, 27.5%) and unemployment (n = 139, 23.2%). The majority of the participants (n = 176, 29.3%) reported an income of less than PKR 30,000 per month in their households. Over half did not have health insurance (n = 323, 53.8%) or a regular health-care provider (n = 307, 51.2%), and most had no physician-diagnosed chronic disease (n = 503, 83.8%). The majority had never smoked (n = 392, 65.3%) or consumed alcohol (n = 460, 76.7%), while most lived in their own homes (n = 238, 39.7%) and relied on salary (n = 227, 37.8%) as their primary household income source.

**Table 2:** Baseline Sociodemographic, Economic, and Health Characteristics of Participants (N = 600) N: number of participants; %: percentage; PKR: Pakistani Rupees. *Other occupation categories include all occupations with small frequencies that were combined into a single category for analysis.

Variable	N	%
Gender		
Male	275	45.8
Female	325	54.2
Marital status		
Single	159	26.5
Married	336	56.0
Divorced	50	8.3
Widowed	23	3.8
Separated	32	5.3
Place of residence		
Urban	401	66.8
Rural	199	33.2
Educational level		
No formal education	73	12.2
Primary education	124	20.7
Secondary education	114	19.0
Higher secondary	109	18.2
Bachelor's degree	83	13.8
Master's degree	56	9.3
MPhil/PhD	41	6.8
Employment status		
Employed (full-time)	165	27.5
Employed (part-time)	62	10.3
Self-employed	97	16.2
Unemployed	139	23.2
Student	27	4.5
Homemaker	72	12.0
Retired	38	6.3
Occupation		
Professional/managerial	35	5.8
Clerical/office	58	9.7
Business owner	63	10.5
Manual/labor	24	4.0
Service/sales	82	13.7
Other occupation categories*	338	56.3
Monthly household income (PKR)		
< 30,000	176	29.3
30,000–59,999	110	18.3
60,000–99,999	101	16.8
100,000–149,999	98	16.3
≥150,000	115	19.2
Health insurance		
No	323	53.8
Yes	277	46.2
Regular healthcare provider		
No	307	51.2
Yes	293	48.8
Physician-diagnosed chronic disease		
No	503	83.8
Yes	97	16.2
Type of chronic disease		
None	503	83.8
Diabetes	12	2.0
Hypertension	19	3.2
Respiratory disease	11	1.8
Cardiovascular disease	8	1.3
Musculoskeletal disease	14	2.3
Multiple/other diseases	33	5.5
Smoking status		
Never smoker	392	65.3
Former smoker	91	15.2
Current smoker	117	19.5
Alcohol consumption		
Never	460	76.7
Occasionally	115	19.2
Regularly	25	4.2
Housing status		
Own house	238	39.7
Rented house	207	34.5
Living with family/relatives	102	17.0
Temporary accommodation	24	4.0
Other	29	4.8
Main household income source		
Salary	227	37.8
Business	97	16.2
Pension	38	6.3
Government assistance	29	4.8
Family support	194	32.3
Other	15	2.5

Table 3 demonstrates that the relationship between the social inequality index and six-month quality of life remained consistently negative and statistically significant across all sensitivity analyses. The primary model showed that higher social inequality was associated with poorer quality of life (β = −0.10, 95% CI: −0.13 to −0.07, p < 0.001). Similar results were observed after removing individual indicators, standardizing the index, categorizing inequality levels, and applying an alternative weighting scheme, with β values ranging from −0.08 to −0.11 and all p-values < 0.001. These findings confirm that the observed association was robust to different methods of index construction.

**Table 3:** Sensitivity Analysis Assessing the Robustness of the Social Inequality Index Association with Six-Month Quality of Life β represents the regression coefficient from the linear mixed-effects model predicting six-month quality of life. Negative coefficients indicate that greater social inequality was associated with poorer quality of life. The sensitivity analyses examined whether the association remained stable under alternative index construction approaches.

Model specification	Index modification	β (SE)	95% CI	p-value
Primary model	Original equally weighted social inequality index	−0.10 (0.02)	−0.13 to −0.07	< 0.001
Sensitivity model 1	Index excluding household income indicator	−0.09 (0.02)	−0.12 to −0.06	< 0.001
Sensitivity model 2	Index excluding education indicator	−0.11 (0.02)	−0.14 to −0.08	< 0.001
Sensitivity model 3	Index excluding housing-related indicator	−0.10 (0.02)	−0.13 to −0.07	< 0.001
Sensitivity model 4	Standardized indicator-based index (z-score transformation before summation)	−0.09 (0.02)	−0.12 to −0.06	< 0.001
Sensitivity model 5	Categorized index (low, moderate, high inequality groups)	−0.08 (0.02)	−0.12 to −0.05	< 0.001
Sensitivity model 6	Index with alternative theoretical weighting scheme	−0.10 (0.02)	−0.13 to −0.06	< 0.001

Table 4 presents the Spearman correlation analysis among the social inequality index, structural vulnerability (SVAT Total), and quality of life (QoL Mean) at baseline for 592 participants. Social inequality was strongly correlated with structural vulnerability (ρ = 0.591, p < 0.001), and higher scores in the social inequality index were associated with higher scores in the structural vulnerability index. Quality of life was also significantly and negatively correlated with social inequality (ρ = −0.530, p < 0.001), indicating that the greater the social disadvantage, the worse the quality of life. Likewise, quality of life showed a strong negative correlation with structural vulnerability (ρ = −0.691, p < 0.001), meaning that higher structural vulnerability was associated with poorer QoL.

**Table 4:** Spearman Correlations Among Social Inequality, Structural Vulnerability, and Quality of Life at Baseline (N = 592) Spearman's rho correlations are reported. SVAT: Structural Vulnerability Assessment Tool; QoL Mean: quality of life mean score; p < 0.01 (two-tailed). The total baseline sample consisted of 600 participants; however, 592 participants were included in the correlation analysis due to missing responses in one or more variables (social inequality index, SVAT Total, or QoL Mean). Missing observations were excluded using pairwise deletion. Higher social inequality index scores indicate greater social disadvantage; higher SVAT Total scores indicate greater structural vulnerability; higher QoL Mean scores indicate better quality of life. Statistical significance was determined at p < 0.001***.

Variable	Social inequality index	SVAT Total	QoL Mean
Social inequality index	—	—	—
SVAT Total	ρ = 0.591, p = < 0.001^***^	—	—
QoL Mean	ρ = −0.530, p = < 0.001^***^	ρ = −0.691, p = < 0.001^***^	—

Table 5 shows the change in HRQoL from baseline to the six-month follow-up. The mean HRQoL score at baseline was 66.70 ± 9.16, which declined to 60.61 ± 10.53 at six months, for a mean score difference of 6.09. The negative change is a sign of a decrease in HRQoL for participants over six months. The differences in HRQoL score were not major at follow-up, although the score differences were somewhat greater, indicating that the mean score for the study participants generally decreased.

**Table 5:** Change in Quality of Life from Baseline to Six-Month Follow-Up HRQoL: health-related quality of life. The mean difference was calculated as the six-month follow-up mean minus the baseline mean. Negative values indicate a decline in health-related quality of life over time. Higher HRQoL scores indicate better quality of life.

Time Point	Mean ± SD	Mean Difference
Baseline	66.70 ± 9.16	—
Six-month follow-up	60.61 ± 10.53	-6.09

Table 6 presents the results of the linear mixed-effects model predicting quality of life over the six-month follow-up. Over time, the quality of life decreased markedly (B = −6.09, p < 0.001). Structural vulnerability (B = −1.48, p < 0.001), a greater number of chronic diseases (B = −2.08, p < 0.001), and higher social inequality (B = −0.10, p < 0.001) were independent factors associated with lower quality of life. In contrast, age (B = 0.02, p = 0.375), gender (B = −0.65, p = 0.686), and place of residence (B = 0.77, p = 0.156) were not significant predictors. Significant interaction effects between time and social inequality (B = 0.03, p = 0.023) and between time and structural vulnerability (B = 0.23, p = 0.001) indicated that the relationship of these factors with quality of life changed over the six-month follow-up period.

**Table 6:** Linear Mixed-Effects Model Predicting Quality of Life Over Six Months B: unstandardized regression coefficient; SE: standard error; CI: confidence interval. The model included participant identification number as a random intercept and time as a repeated measure. Higher social inequality index scores indicate greater social disadvantage. Higher Structural Vulnerability Assessment Tool total scores indicate greater structural vulnerability. Higher Quality of Life scores indicate better quality of life. *p < 0.05, **p < 0.01, ***p < 0.001 indicate statistically significant results.

Predictor	B	SE	df	t	p	95% CI
Intercept	87.17	1.97	620.98	44.23	< 0.001^***^	83.30 - 91.04
Time (baseline vs. 6 months)	−6.09	0.61	589	−9.94	< 0.001^***^	−7.29 - −4.88
Social inequality index	−0.10	0.02	604.25	−5.83	< 0.001^***^	−0.13 -−0.07
Structural Vulnerability Assessment Tool total score	−1.48	0.08	587.24	−18.48	< 0.001^***^	−1.63 -−1.32
Age	0.02	0.02	583.96	0.89	0.375	−0.02 - 0.06
Gender	−0.65	1.61	583.96	−0.41	0.686	−3.80 - 2.50
Residence	0.77	0.54	583.96	1.42	0.156	−0.30 - 1.84
Number of chronic diseases	−2.08	0.43	583.96	−4.87	< 0.001^***^	−2.92 - −1.24
Time × Social inequality index	0.03	0.01	589	2.29	0.023^*^	0.004 - 0.06
Time × Structural Vulnerability Assessment Tool total score	0.23	0.07	589	3.48	0.001^**^	0.10 - 0.36

Table 7 presents the baseline-adjusted predictors of six-month quality of life using ANCOVA. Baseline quality of life was a strong positive predictor of six-month QoL (B = 0.664, p < 0.001, partial η² = 0.381). The effects of greater structural vulnerability were independent in predicting poor quality of life at six months (B = −0.658, p < 0.001, partial η² = 0.112). In contrast, social inequality (B = 0.626, p = 0.445), age (B = −0.018, p = 0.375), household size (B = −0.433, p = 0.456), financial dependents (B = −0.282, p = 0.639), and the number of chronic diseases (B = −0.620, p = 0.125) were not significant predictors of six-month QoL after adjustment. The difference between the mixed-effects and ANCOVA findings for social inequality reflects the different analytical objectives of the two models. The mixed-effects model evaluated longitudinal patterns across repeated measurements and included the time-by-social inequality interaction, whereas ANCOVA focused on the six-month outcome after adjustment for baseline HRQoL. Therefore, the findings represent different aspects of the relationship between social inequality and HRQoL rather than directly contradictory results.

**Table 7:** Baseline-Adjusted Predictors of Six-Month Quality of Life: Univariate Analysis of Covariance The dependent variable was the six-month Quality of Life mean score. B: unstandardized regression coefficient; SE: standard error; CI: confidence interval; Partial η²: partial eta squared. The model was adjusted for baseline Quality of Life, social inequality index, Structural Vulnerability Assessment Tool total score, age, household size, financial dependents, and number of chronic diseases. Higher Structural Vulnerability Assessment Tool total scores indicate greater structural vulnerability. Higher Quality of Life scores indicate better quality of life. Statistical significance was determined at p < 0.001***.

Predictor	B	SE	t	p	95% CI	Partial η²
Baseline Quality of Life	0.664	0.035	18.71	< 0.001^***^	0.594 - 0.734	0.381
Social inequality index	0.626	0.819	0.76	0.445	−0.983 - 2.235	0.001
Structural Vulnerability Assessment Tool total score	−0.658	0.078	−8.48	< 0.001^***^	−0.810 - −0.505	0.112
Age	−0.018	0.020	−0.89	0.375	−0.058 - 0.022	0.001
Household size	−0.433	0.581	−0.75	0.456	−1.575 - 0.708	0.001
Financial dependents	−0.282	0.601	−0.47	0.639	−1.463 - 0.898	< 0.001
Number of chronic diseases	−0.620	0.404	−1.53	0.125	−1.414 - 0.174	0.004

Table 8 shows the effects of the categorical factors (sociodemographic and health-related factors) on 6-month post-baseline QOL after controlling for baseline QOL and other covariates. None of the examined factors, including gender [F(1,569) = 1.76, p = 0.186], residence [F(1,569) = 0.92, p = 0.339], educational level [F(6,569) = 0.60, p = 0.733], employment status [F(6,569) = 1.85, p = 0.087], household income [F(4,569) = 0.37, p = 0.834], housing status [F(4,569) = 0.37, p = 0.830], or health insurance [F(1,569) = 0.75, p = 0.388], showed a statistically significant association with six-month QoL. All effect sizes were small (partial η² ≤ 0.019), and these factors accounted for a relatively small proportion of the variance in quality of life outcomes.

**Table 8:** Effects of Sociodemographic and Health-Related Factors on Six-Month Quality of Life The table presents the results of the univariate analysis of variance for categorical predictors of six-month Quality of Life. The dependent variable was the six-month Quality of Life mean score. F: F statistic; df: degrees of freedom; η²p: partial eta squared (effect size). The model was adjusted for baseline Quality of Life, social inequality index, Structural Vulnerability Assessment Tool total score, age, household size, financial dependents, and number of chronic diseases. Higher η²p values indicate greater effect size. Statistical significance was determined at p < 0.05.

Factor	F(df1, df2)	p	η²p
Gender	1.76(1, 569)	0.186	0.003
Residence	0.92(1, 569)	0.339	0.002
Education	0.60(6, 569)	0.733	0.006
Employment	1.85(6, 569)	0.087	0.019
Household income	0.37(4, 569)	0.834	0.003
Housing status	0.37(4, 569)	0.830	0.003
Health insurance	0.75(1, 569)	0.388	0.001

## Discussion

This prospective cohort study found that structural vulnerability remained a significant independent predictor of poorer HRQoL at six-month follow-up. At the same time, the relationship between social inequality and HRQoL was no longer significant after accounting for baseline HRQoL and other relevant baseline covariates. Longitudinal analyses also showed that structural vulnerability remained an independent predictor of HRQoL. In contrast, the impact of social inequality was reduced when HRQoL at baseline and other baseline variables were included as covariates. These findings align with previous studies showing that socioeconomic disadvantage and structural barriers are associated with poorer HRQoL [12-14]. But, unlike previous studies, our longitudinal analyses suggested that only structural vulnerability was an independent predictor of HRQoL after adjustment, with social inequality having a weaker effect. They suggest that structural vulnerability may be a more comprehensive measure of social disadvantage associated with HRQoL over the six-month follow-up period. This might be because structural vulnerability measures a wider spectrum of interrelated social and structural disadvantages, such as financial insecurity, unstable housing, lack of access to health care, and lack of social support. In contrast, traditional socioeconomic measures only capture individual dimensions of disadvantage. Thus, structural vulnerability could offer a more complete indicator of the cumulative social factors associated with HRQoL over the six-month follow-up period.

Another interesting result from this study was the high positive relationship between social inequality and structural vulnerability, where people who were more socially disadvantaged were also more likely to be more structurally vulnerable with respect to financial insecurity, substandard housing, lack of healthcare access, and less social support. This is consistent with earlier work establishing that social vulnerability is strongly associated with socioeconomic adversity and is multi-dimensional, defined by measures such as income, housing, health, and social supports. People living with higher socioeconomic disadvantage are therefore more likely to encounter multiple structural barriers that have negative health and wellbeing implications [5, 15]. In addition, higher levels of social inequality and structural vulnerability were strongly correlated with poorer baseline HRQoL, indicating that adverse social factors were associated with poorer physical, psychological, social, and environmental functioning. These results corroborate earlier studies showing that other social factors, such as socioeconomic status, significantly influence HRQoL in addition to medical conditions [4, 13]. The results indicate that social disadvantage may be associated with poorer quality of life through pathways involving decreased access to health care, increased social and psychosocial stress, financial strain, and weak social support, which can harm the physical, psychological, social, and environmental aspects of well-being.

The longitudinal analyses provide further insight into these relationships. Quality of life worsened markedly during the follow-up period, and both structural vulnerability and the number of chronic diseases independently predicted poorer HRQoL. These results are consistent with earlier longitudinal studies showing that social disadvantage, unmet social needs, and multimorbidity are factors associated with worse HRQoL over time. The substantial interaction effects between time and social inequality and structural vulnerability, however, indicate that the role of these social determinants may change over time, adding to existing evidence that they might have a dynamic association with HRQoL over the six-month follow-up period [16-18]. Although social inequality was significantly associated with HRQoL in the mixed-effects model, it was no longer an independent predictor after adjustment in the ANCOVA model. This aligns with the concept of structural vulnerability as a multi-dimensional phenomenon that encompasses a wider range of structural determinants, such as housing, health care, financial insecurity, and social support, in addition to more classical socioeconomic factors [15, 8]. Thus, structural vulnerability may better capture the pathways of socioeconomic disadvantage on quality of life. The contrasting findings for social inequality across the two models should be interpreted in the context of their different analytical frameworks. The mixed-effects model identified a significant association between social inequality and HRQoL over the repeated measurements. In contrast, ANCOVA did not identify an independent association with six-month HRQoL after adjustment for baseline HRQoL. This difference may reflect the longitudinal nature and time interaction captured by the mixed-effects model compared with the baseline-adjusted single follow-up outcome assessed by ANCOVA. Thus, the two analyses provide complementary, not contradictory, information about the association between social inequality and HRQoL.

Demographic variables like gender, education, employment status, household income, residence, housing status, and health insurance were not independently associated with 6-month HRQoL after adjustment. These results suggest that the cumulative burden of structural disadvantage may influence quality of life more than individual demographic characteristics considered separately. Previous studies have shown that cumulative social and neighborhood disadvantage correlates better with health and HRQoL than single sociodemographic or cross-sectional measures of disadvantage [19-21]. This highlights the need for a multi-dimensional perspective to measure and identify the individuals and populations most vulnerable to poor health outcomes when examining the social determinants of health [22, 23].

These findings have meaningful implications for healthcare practice and public health policy. To improve health-related quality of life, interventions must go beyond clinical management and address structural factors that affect health. Regular structural vulnerability assessments in health care settings could help identify people at higher social risk and enable timely referrals to social and community resources. Health policies that increase access to health services, enhance social protection measures, ease financial constraints, and tackle social inequalities more comprehensively can play a significant role in improving QoL among vulnerable groups over time.

The strengths of this study include its prospective cohort design, relatively large sample size with minimal loss to follow-up, simultaneous assessment of social inequality and structural vulnerability, and use of validated instruments, including the Structural Vulnerability Assessment Tool and WHOQOL-BREF. In addition, repeated quality-of-life assessments using a longitudinal mixed-effects modelling approach yielded greater evidence than cross-sectional analyses alone, as it accounted for within-subject variability.

Limitations

There are several drawbacks to recognize. First, convenience sampling was used to recruit participants from healthcare facilities and community-based sites in Karachi, which may have introduced selection bias and limited generalizability. Recruitment through healthcare facilities may have preferentially included individuals with existing health concerns, greater healthcare needs, or better access to healthcare services. In contrast, recruitment from community settings may have captured individuals with different socioeconomic and healthcare-access profiles. Consequently, the distribution of social inequality index and structural vulnerability scores in our sample may differ from that of the broader adult population. Individuals with the greatest structural vulnerability may also have been less likely to access or participate in healthcare-based recruitment, potentially resulting in underrepresentation of the most disadvantaged groups. Second, the observational design limits the ability to make strong causal inferences, despite the advantages of the prospective cohort design. Third, the authors evaluated social inequality and structural vulnerability only at baseline and did not assess changes in these factors during follow-up. Additionally, the six-month follow-up period was relatively short and may not capture longer-term changes in social inequality, structural vulnerability, or HRQoL. Fourth, the study used self-reported questionnaires, which may introduce recall and social desirability bias. The social inequality index was constructed specifically for this study as an equally weighted composite of selected socioeconomic and social indicators and has not undergone formal psychometric validation. Although equal weighting provides a transparent, non-subjective approach, it assumes each component contributes equally to overall social disadvantage, which may not reflect their actual relative importance. Therefore, interpret the index as a pragmatic multidimensional indicator rather than a validated measurement instrument. Future studies should evaluate its reliability, construct validity, and alternative weighting approaches in larger and more diverse populations. Lastly, although several demographic and health-related factors have been adjusted for, it is never possible to rule out residual confounding from unmeasured psychosocial or health-related factors.

Future directions

Future studies should include nationally representative multicentre cohorts with longer follow-up periods to better understand the long-term effects of social inequality and structural vulnerability on HRQoL. Multiple evaluations of social situation over time would help to determine the effect of changes in structural vulnerability on health outcomes. Additionally, intervention studies examining policies and community-based programmes to mitigate structural vulnerability should be conducted to see whether improvements in these upstream social determinants result in durable improvements in QoL.

## Conclusions

This prospective cohort study found that structural vulnerability was significantly associated with HRQoL in adults. While greater social inequality was associated with poorer HRQoL, structural vulnerability remained the strongest independent predictor of HRQoL at six months after adjustment for social inequality and other relevant covariates. These findings highlight the importance of considering structural and social factors alongside clinical care when addressing health inequities and improving HRQoL. Incorporating assessment of structural vulnerability into healthcare practice and public health planning may help identify socially disadvantaged groups and support more equitable health outcomes.
